# Culture–gene coevolution of individualism–collectivism and the serotonin transporter gene

**DOI:** 10.1098/rspb.2009.1650

**Published:** 2009-10-28

**Authors:** Joan Y. Chiao, Katherine D. Blizinsky

**Affiliations:** 1 Department of Psychology, Northwestern University, Evanston, IL, USA; 2 Interdepartmental Neuroscience Program, Northwestern University, Evanston, IL, USA

**Keywords:** culture–gene coevolution, serotonin transporter gene, 5-HTTLPR, mood disorders, individualism–collectivism, cultural neuroscience

## Abstract

Culture–gene coevolutionary theory posits that cultural values have evolved, are adaptive and influence the social and physical environments under which genetic selection operates. Here, we examined the association between cultural values of individualism–collectivism and allelic frequency of the serotonin transporter functional polymorphism (5-HTTLPR) as well as the role this culture–gene association may play in explaining global variability in prevalence of pathogens and affective disorders. We found evidence that collectivistic cultures were significantly more likely to comprise individuals carrying the short (S) allele of the 5-HTTLPR across 29 nations. Results further show that historical pathogen prevalence predicts cultural variability in individualism–collectivism owing to genetic selection of the S allele. Additionally, cultural values and frequency of S allele carriers negatively predict global prevalence of anxiety and mood disorder. Finally, mediation analyses further indicate that increased frequency of S allele carriers predicted decreased anxiety and mood disorder prevalence owing to increased collectivistic cultural values. Taken together, our findings suggest culture–gene coevolution between allelic frequency of 5-HTTLPR and cultural values of individualism–collectivism and support the notion that cultural values buffer genetically susceptible populations from increased prevalence of affective disorders. Implications of the current findings for understanding culture–gene coevolution of human brain and behaviour as well as how this coevolutionary process may contribute to global variation in pathogen prevalence and epidemiology of affective disorders, such as anxiety and depression, are discussed.

## 1. Introduction

Conventional evolutionary biology theory posits that organisms adapt to their environment and over time exhibit favourable traits or characteristics that best enable them to survive and reproduce in their given environment through the process of natural selection (Darwin 1859). The concept of natural selection has been enormously influential to the study of human behaviour, particularly in evolutionary psychology, which has emphasized that much of human behaviour arises as a by-product of adaptive mechanisms in the mind and brain (Barkow *et al*. 1992). More recently, culture–gene coevolution has emerged as an influential theory to explain how human behaviour is a product of two complementary and interacting evolutionary processes: genetic and cultural evolution (Cavalli-Sforza & Feldman 1981; Lumsden & Wilson 1981; Boyd & Richerson 1985). This dual inheritance theory of human behaviour proposes that cultural traits are adaptive and they evolve and influence the social and physical environments under which genetic selection operates (Boyd & Richerson 1985). A prominent example of the dual inheritance theory is the culture–gene coevolution between cattle milk protein genes and human lactase genes (Beja-Pereira *et al*. 2003), whereby the cultural propensity for milk consumption in humans has led to genetic selection for milk protein genes in cattle and gene encoding lactase in humans. Although well studied with computational modelling approaches (Smith *et al*. 2008), the study of culture–gene coevolutionary theory of human behaviour has not yet received widespread empirical attention.

**Figure 1.**
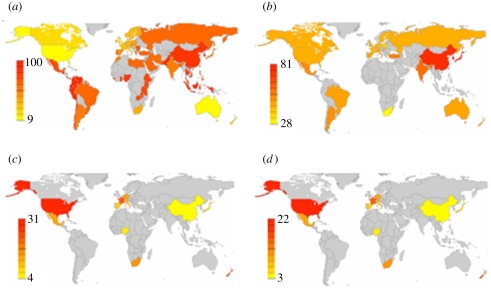
Geographical coincidence between serotonin transporter gene diversity and cultural traits of individualism–collectivism across countries. Colour maps include all available published data for each variable of interest. Grey areas indicate geographical regions where no published data are available. (*a*) Colour map of frequency distribution of IND-COL from Hofstede (2001). (*b*) Colour map of frequency distribution of S alleles of 5-HTTLPR. (*c*) Colour map of frequency of global prevalence of anxiety. (*d*) Colour map of frequency of global prevalence of mood disorders. Yellow to red colour bar indicates low to high prevalence.

A fundamental way in which culture shapes human behaviour is through self-construal style, or in how people define themselves and their relation to others in their environment (Markus & Kitayama 1991; Triandis 1995; Nisbett *et al*. 2001). In particular, cultural psychologists have identified two primary styles of self-construal across cultures: individualism and collectivism (Markus & Kitayama 1991; Triandis 1995; Nisbett *et al*. 2001) (figure 1*a*). Individualistic cultures encourage thinking of people as independent of each other. By contrast, collectivistic cultures endorse thinking of people as highly interconnected to one another. Individualistic cultures emphasize self-expression and pursuit of individuality over group goals, whereas collectivistic cultures favour maintenance of social harmony over assertion of individuality (Markus & Kitayama 1991; Triandis 1995; Nisbett *et al*. 2001). Self-construal style affects a wide range of human behaviour, including how people feel, think, perceive and reason about people and objects in their environment (Nisbett *et al*. 2001; Kitayama & Cohen 2007), and their underlying neural substrates (Chiao & Ambady 2007; Chiao in press). Evident from the writings of Socrates and Lao Tzu, cultural divergences in ancient Western and East Asian philosophical views of the self are thought to have emerged early in human history (Markus & Kitayama 1991; Triandis 1995; Nisbett *et al*. 2001). However, a parsimonious explanation of the origin of individualistic and collectivistic cultural values has largely remained elusive.

In addition to cultural factors, human behaviour is influenced by specific genes, such as the serotonin transporter gene (*SLC6A4*), which regulates serotonergic neurotransmission (5-HTT) (Lesch *et al*. 1996; Canli & Lesch 2007). The serotonin transporter gene (*SLC6A4*) contains a polymorphic region, known as 5-HTTLPR, comprising a short (S) allele and a long (L) allele version that results in differential 5-HTT expression and function (Lesch *et al*. 1996; Hariri 2009). Individuals carrying the S allele of the 5-HTTLPR produce significantly less 5-HTT mRNA and protein, resulting in higher concentrations of serotonin in the synaptic cleft relative to individuals carrying the L allele (Lesch *et al*. 1996). Evidence from behavioural genetics indicates that the S allele of the serotonin transporter gene (5-HTTLPR) is associated with increased negative emotion, including heightened anxiety (Sen *et al*. 2004; Munafò *et al*. 2005), harm avoidance (Munafò *et al*. 2005), fear conditioning (Lonsdorf *et al*. 2009), attentional bias to negative information as well as increased risk for depression in the presence of environmental risk factors (Caspi *et al*. 2003; Taylor *et al*. 2006; Uher & McGuffin 2008; see also Munafò *et al*. 2009). In particular, exposure to chronic life stress, such as interpersonal conflict, loss or threat, is considered a well-known environmental risk factor for depression in S allele carriers of the 5-HTT (Caspi *et al*. 2003; see also Risch *et al*. 2009). Convergent evidence from endophenotypes indicates that activity in brain regions that are regulated by serotonergic neurotransmission and are critical to emotional behaviour, such as the amygdala, varies as a function of 5-HTT. Specifically, individuals carrying the S allele show greater amygdala response (Hariri *et al*. 2002; Munafò *et al*. 2008), which is likely due to increased amygdala resting activation (Canli *et al*. 2005) and decreased functional coupling between the amygdala and subgenual cingulate gyrus (Pezawas *et al*. 2005), relative to those carrying the L allele.

Evidence from population genetics reveals greater population frequency of 5-HTTLPR S allele carriers of the 5-HTTLPR functional polymorphism within certain geographical regions of the world, such as East Asia (figure 1*b*). In a typical East Asian sample, 70–80% of individuals are S carriers compared with a typical European sample where 40–45% of individuals are S carriers of the 5-HTT genotype (Gelernter *et al*. 1997; Nakamura *et al*. 1997). It remains unclear why there exists genetic selection for S relative to L allele carriers in East Asian regions, but not other geographical regions of the world. One possible explanation for greater prevalence of S allele carriers in East Asia is that geographical variability in environmental pressures has led to cultural variability in individualism–collectivism via genetic selection. Recent research has shown that geographical variability in historical and contemporary pathogen prevalence predicts variability in individualistic and collectivistic cultural norms (Fincher *et al*. 2008). That is, nations with greater historical and contemporary prevalence of disease-causing pathogens or infectious diseases (e.g. malaria, typhus and leprosy) are more likely to endorse collectivistic cultural norms, likely due to the anti-pathogen defence function that collectivistic norms may serve. Given the adaptive value of collectivistic cultural values, it is possible that increased pathogen prevalence in East Asian regions may be associated with increased collectivistic values due to genetic selection of the S allele of the serotonin transporter gene within collectivistic cultures.

Additionally, based on prior evidence from behavioural genetics studies conducted in Western populations, one might suspect a heightened prevalence of negative affect and related disorders in East Asian populations given the greater prevalence of individuals carrying the S allele. On the contrary, evidence from a number of cross-cultural epidemiological studies indicates that East Asian populations consistently report lower prevalence of negative affect, such as anxiety (Kessler & Ustun 2008) and mood disorders (e.g. major depressive disorder and bipolar disorder) (Weissman *et al*. 1996; Kessler & Ustun 2008), relative to Western populations (figure 1*c*,*d*, respectively). It remains unclear why anxiety and mood disorders are less prevalent in East Asian relative to Western cultures, especially given that a majority of individuals living in East Asia carry the S allele of the serotonin transporter gene, which is associated in Western populations with negative affect.

A potentially parsimonious explanation for the increased prevalence of S allele carriers, yet decreased prevalence of anxiety and mood disorders, in East Asia relative to other geographical regions is culture–gene coevolution of human behaviour. Culture–gene coevolutionary theory proposes that cultural traits, such as individualism and collectivism, have evolved and are adaptive. Supporting the notion of cultural traits as evolutionary adaptations, recent cross-national evidence shows that cultural values of individualism and collectivism serve an adaptive, ‘anti-pathogen’ function, protecting vulnerable geographical regions from increased spread of disease-causing pathogens via the promotion of collectivistic social norms, such as conformity and parochialism (Fincher *et al*. 2008). Similarly, here we propose that by favouring social harmony over individuality, collectivistic cultural norms may have evolved to also serve an adaptive, ‘anti-psychopathology’ function, creating an environmental niche that reduces the risk of exposure to environmental pathogens, such as chronic life stress, for group members. Consistent with a gene-by-environment (GxE) theory of affective disorders, reduced exposure to chronic life stress for individuals living in collectivistic relative to individualistic cultures would then cause reduced prevalence of affective disorders among genetically susceptible individuals. Hence, culture variation in the epidemiological prevalence of anxiety and depression is likely due to geographical variation in the cultural adoption of collectivistic social norms.

Here, we test this culture–gene coevolution hypothesis by examining the association between the serotonin transporter gene, individualism–collectivism and prevalence of anxiety and mood disorders across nations. Specifically, we hypothesized that increased frequency of S allele carriers of the 5-HTTLPR functional polymorphism within East Asia is due to culture–gene coevolution, whereby collectivistic cultural values serve an adaptive function, reducing the probability of environmental stress, a known catalyst of negative affect, thus leading to genetic selection of the S allele within collectivistic cultures. Analyses were conducted using aggregate published data on allelic frequency of 5-HTTLPR, cultural values of individualism–collectivism and global prevalence of anxiety and mood disorders, which refers to bipolar disorder, dysthymia and major depressive disorder defined by DSM IV/CIDI criteria in the 2008 World Health Organization (WHO) survey, with nation as the cultural unit of analysis.

Additionally, given prior evidence that geographical variability in pathogen prevalence is associated with cultural variability in individualism–collectivism, we also examined the association between the serotonin transporter gene, individualism–collectivism and prevalence of disease-causing pathogens across nations. We hypothesized that increased pathogen prevalence is associated with increased collectivistic values due to genetic selection of the S allele of the serotonin transporter gene within collectivistic cultures. Analyses were conducted using aggregate published data on allelic frequency of 5-HTTLPR, cultural values of individualism–collectivism and global prevalence of historical and contemporary pathogen prevalence, with nation as the cultural unit of analysis.

## 2. Material and methods

### (a) Cross-national sample of allelic frequency of 5-HTTLPR

Data on allelic frequency of the 5-HTTLPR from 50 135 individuals living in 29 countries (Argentina, Australia, Austria, Brazil, Denmark, Estonia, Finland, France, Germany, Hungary, India, Israel, Italy, Japan, Korea, Mexico, the Netherlands, New Zealand, Poland, People's Republic of China, Russia, South Africa, Singapore, Spain, Sweden, Taiwan, Turkey, UK and USA) were compiled from 124 peer-reviewed publications (see table S1 and methods in electronic supplementary material for further detail).

### (b) Cross-national sample of cultural values

Given evidence of strong correlations between independent measures of individualism and collectivism (*r* ≥ 0.80) (Fincher *et al*. 2008), we used Hofstede's published regional scores of individualism–collectivism across 29 nations (Argentina, Australia, Austria, Brazil, Denmark, Estonia, Finland, France, Germany, Hungary, India, Israel, Italy, Japan, Korea, Mexico, the Netherlands, New Zealand, Poland, People's Republic of China, Russia, South Africa, Singapore, Spain, Sweden, Taiwan, Turkey, UK and USA). Additionally, we used Hofstede's scores of power distance, uncertainty avoidance, masculinity–femininity and long-term orientation across 22 nations (see table S1 and methods in electronic supplementary material).

### (c) Cross-national sample of economic indices

Owing to the possible association between economic indices, such as gross domestic product (GDP) and Gini index, an index of inequality in income distribution, and cultural values of individualism and collectivism, we included data from these two economic indices in analyses for 29 countries (see table S1 and methods in electronic supplementary material).

### (d) Cross-national sample of pathogen prevalence

Owing to the known prior association between pathogen prevalence and the cultural value of individualism–collectivism, we included data of both contemporary and historical pathogen prevalence in multiple regression analyses and mediation analyses (see electronic supplementary material, table S1).

### (e) Cross-national sample of prevalence of mental health disorders

Data on global prevalence of mental health disorders, including anxiety, mood disorder, impulse control and substance abuse, were compiled from the 2008 WHO Mental Health Surveys Report (Kessler & Ustun 2008). Using all available data, there were a total of 12 nations included in the regression of mediation analysis (France, Germany, Israel, Italy, Japan, Mexico, the Netherlands, New Zealand, People's Republic of China, South Africa, Spain and USA; see table S1 and methods in electronic supplementary material).

### (f) Statistical analysis

Standard multiple regression and mediation analytic techniques were used to explore the relationship between cultural traits of individualism–collectivism, allelic frequency of the 5-HTTLPR serotonin transporter gene and global prevalence of pathogens and mental health disorders (see methods in electronic supplementary material). There was heterogeneity of sample size (e.g. number of nations where data were available) across the different variables of interest due to limitations in data availability across the Hofstede cultural value indices, 5-HTTLPR allelic frequency, pathogen prevalence and 2008 WHO Report datasets. In order to maximize the sample size of each analysis, we included all available published data for each variable in the multiple regression and mediation regression analyses.

### (g) Nation as unit of analysis

For all primary analyses, geographical regions defined by nation served as the unit of analysis given that numerous prior studies have shown that geopolitical regions are reliable proxies of societal cultures (Schwartz 2004; Fincher *et al.* 2008). Similar to Fincher *et al*. (2008), in addition to our primary analyses that focused on nation as the primary unit of analysis, we also conducted correlational analyses of prevalence of the S allele and cultural values of individualism–collectivism with Murdock's (1949) six world regions and Gupta & Hanges' (2004) 10 distinct cultural clusters as the primary unit of analysis (see methods in electronic supplementary material).

## 3. Results

**Figure 2.**
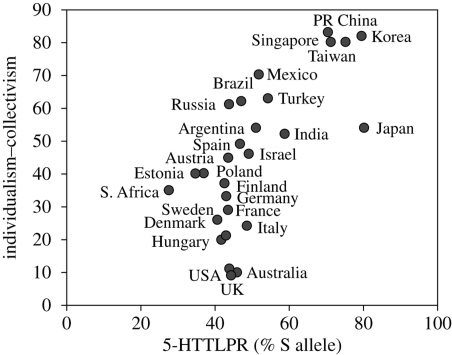
Results from correlation analysis between Hofstede's individualism–collectivism index (reverse scored) and frequency of S allele carriers of the 5-HTTLPR across 29 nations. Collectivistic nations showed higher prevalence of S allele carriers (*r*(29) = 0.70, *p* < 0.0001).

First, we assessed the global association between 5-HTTLPR and cultural values of individualism–collectivism. Collectivistic cultures were significantly more likely to comprise individuals carrying the S allele of the 5-HTTLPR (*r*(29)=0.70, *p* < 0.0001) (figure 2). This strong correlation observed between prevalence of the S allele and cultural values of individualism–collectivism is replicated when broader cultural regions are treated as the units of analysis. Irrespective of whether the world is partitioned into Murdock's six world regions (*r*(6)=0.85, *p* < 0.02) or Gupta and Hanges' 10 distinct cultural clusters (*r*(9)=0.67, *p* < 0.03), increased collectivism was positively and significantly correlated with increased prevalence of S alleles.

To determine the specificity of the association between 5-HTTLPR and individualism–collectivism, we conducted a multiple regression analysis with population frequency of S allele carriers as the criterion variable and cultural values of individualism–collectivism as well as four other well-known cultural values, specifically power distance, uncertainty avoidance, masculinity–femininity and long–short-term orientation, as predictor variables. Results indicated that the cultural value of individualism–collectivism was the only significant predictor of the frequency of S allele carriers of 5-HTTLPR across 22 nations (*β* = 0.52, *p* < 0.02) (table 1).

**Table 1.** Results from multiple regression analyses examining the association between cultural values of individualism–collectivism and the serotonin transporter gene across nations. All available published data for each variable were included in the regression analyses. Different numbers of included cases in each analysis reflect differences in the amount of available published data (e.g. number of nations) for each variable of interest. *β*, Standardized beta coefficients. Bold values indicate significant variables.

criterion variable	predictor variables	*β*	*t*	*p*-value
% S allele (*n* = 22)	**IND-COL**	**0.52**	**2.60**	<0.02*
	power distance	0	0.03	=0.98
	uncertainty avoidance	0.12	0.92	=0.37
	masculinity–femininity	−0.20	−1.43	=0.17
	long-term/short-term orientation	−0.39	−2.07	=0.06
IND-COL (*n* = 29)	**% S allele**	**0.61**	**3.58**	<0.002**
	GDP	−0.27	−1.4	=0.17
	Gini index	0.15	0.87	=0.39
	pathogen contemporary	0.13	0.11	=0.92
	pathogen historical	−0.03	−0.54	=0.59

*p < 0.05.

**p < 0.005.

Second, we sought to determine whether the frequency of S allele carriers predicts cultural individualism and collectivism by conducting a multiple regression analysis with individualism–collectivism as the criterion variable and frequency of S allele carriers, as well as four other economic and health factors previously associated with individualism–collectivism including GDP *per capita*, inequity in the distribution of wealth (Gini index) as well as historical and contemporary pathogen prevalence as predictor variables (Fincher *et al*. 2008). Results indicate that allelic frequency of S carriers was the only significant predictor of individualism–collectivism across 29 nations (*β* = 0.61, *p* < 0.002) (table 1).

Third, we conducted regression of mediation to test the hypothesis that the frequency of S allele carriers is negatively associated with negative affect, such as anxiety (electronic supplementary material, figure S1*a*) and mood disorders (electronic supplementary material, figure S1*b*), across cultures because of the buffering effects of the cultural values of individualism–collectivism. In the first step, we sought to determine whether 5-HTTLPR is associated with cultural values of individualism–collectivism as well as anxiety and mood disorders across nations. Consistent with our earlier finding using a larger dataset, across 12 nations, the frequency of S allele carriers of the 5-HTTLPR was a significant positive predictor of cultural values of individualism–collectivism (*β* = 0.94, *p* < 0.05). Additionally, across 12 nations, the frequency of S allele carriers of the 5-HTTLPR was a significant negative predictor of anxiety (*β* = −0.31, *p* < 0.05) and mood disorders (*β* = −0.21, *p* < 0.05). Nations with a higher frequency of S allele carriers showed a lower prevalence of anxiety (*r*(12) = −0.55, *p* < 0.03) and mood disorders (*r*(12) = −0.52, *p* < 0.05). In the second step of the mediation analysis, we examined whether cultural values of individualism–collectivism were associated with anxiety (electronic supplementary material, figure S1*c*) and mood disorders (electronic supplementary material, figure S1*d*) across cultures. Across 12 nations, individualism–collectivism was a significant negative predictor of anxiety (*β* = −0.73, *p* < 0.008) and mood disorder (*β* = −0.80, *p* < 0.002) prevalence. Collectivistic nations showed a lower prevalence of anxiety (*r*(12) = −0.73, *p* < 0.004) and mood disorders (*r*(12) = −0.80, *p* < 0.001) (table 2).

**Table 2.** Results from the mediation regression analysis examining the relationship between cultural values of individualism–collectivism, the serotonin transporter gene and affective disorders across nations. All available published data for each variable were included in the regression analyses. Different numbers of included cases in each analysis reflect differences in the amount of available published data (e.g. number of nations) for each variable of interest. *β*, Standardized beta coefficients. Bold values indicate significant variables.

criterion variable	predictor variables	*β*	*t*	*p*-value
anxiety (*n* = 12)	% S allele	−0.08	−0.49	=0.63
	**IND-COL**	**−0.24**	**−2.15**	<0.03*
mood disorder (*n* = 12)	% S allele	−0.02	−0.08	=0.94
	**IND-COL**	**−0.79**	**−3.01**	<0.02*
impulse control (*n* = 9)	% S allele	0.12	0.25	=0.81
	IND-COL	−0.56	−1.12	=0.30
substance abuse (*n* = 12)	% S allele	−0.35	−0.96	=0.36
	IND-COL	−0.24	−0.64	=0.54

*p < 0.05.

**Figure 3.**
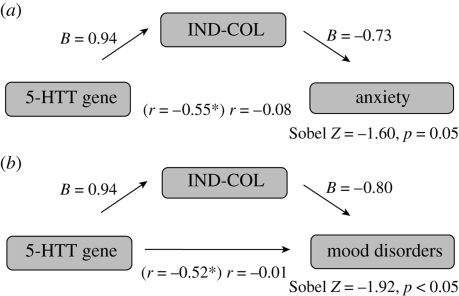
Illustration of mediation analyses between S allele frequency of 5-HTTLPR, individualism–collectivism and global prevalence of anxiety and mood disorders across 12 nations. (*a*) In the mediation regression where both S allelic frequency and cultural values of individualism–collectivism were included as predictors of global anxiety prevalence across 12 nations, individualism–collectivism remained a reliable predictor (*β* = −0.26, *p* < 0.05), and the effect of S allele frequency decreased significantly (from *r*(12) = −0.55 to *r*(12) = −0.16; Sobel test *Z* = −1.60, *p* = 0.05). Asterisk indicates a significant value. (*b*) In the mediation regression where both S allelic frequency and cultural value of individualism–collectivism were included as predictors of global depression prevalence across 12 nations, individualism–collectivism remained a reliable predictor (*β* = −0.21, *p* < 0.05), and the effect of S allele frequency decreased significantly (from *r*(12) = −0.51 to *r*(12) = −0.03; Sobel test *Z* = −1.92, *p* < 0.05). Asterisk indicates a significant value.

In the mediation regression where both S allelic frequency and cultural values of individualism–collectivism were included as predictors of global anxiety prevalence across 12 nations, individualism–collectivism remained a reliable predictor (*β* = −0.24, *p* < 0.05), and the effect of S allele frequency decreased significantly (from *r*(12) = −0.55 to *r*(12) = −0.08; Sobel test *Z* = −1.60, *p* = 0.05) (figure 3*a*). Similarly, in the mediation regression where both S allelic frequency and cultural value of individualism–collectivism were included as predictors of global depression prevalence across 12 nations, individualism–collectivism remained a reliable predictor (*β* = −0.23, *p* < 0.05), and the effect of S allele frequency decreased significantly (from *r*(12) = −0.51 to *r*(12) = −0.01; Sobel test *Z* = −1.92, *p* < 0.05) (figure 3*b*).

To further determine the specificity of the mediating role of collectivistic cultural values on the association between allelic frequency of the 5-HTTLPR and mental health disorders, we also conducted regression of mediation on 5-HTTLPR, individualism–collectivism and two mental health disorders included in the 2008 WHO Mental Health Survey, but not previously associated with the 5-HTTLPR, namely substance abuse and impulse control. As predicted, neither frequency of S allele carriers of the 5-HTTLPR nor cultural values of individualism–collectivism was significantly associated with either impulse control across nine nations or substance abuse across 12 nations (all *p* > 0.05) (table 2). Taken together, the current findings support our hypothesis that population frequency of S allele carriers predicts decreased prevalence of anxiety and mood disorders across nations owing to increased collectivistic cultural values.

**Figure 4.**
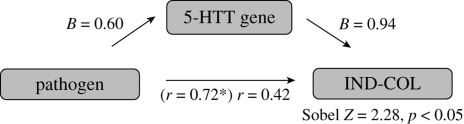
Illustration of mediation analyses between historical pathogen prevalence, S allele frequency of 5-HTTLPR and cultural values of individualism–collectivism across 29 nations. In the mediation regression where both historical pathogen prevalence and S allelic frequency were included as predictors of cultural values of individualism–collectivism across 29 nations, S allelic frequency remained a reliable predictor (*β* = 0.45, *p* < 0.007), and the effect of global historical pathogen prevalence decreased significantly (from *r*(29) = 0.72 to *r*(29) = 0.42; Sobel test *Z* = 2.28, *p* < 0.05). Asterisk indicates a significant value.

Finally, we conducted regression of mediation to test an additional hypothesis that global historical and contemporary pathogen prevalence is positively associated with cultural values of individualism–collectivism across cultures because of genetic selection for the S allele of the serotonin transporter gene. We found that global historical (*β* = 0.60, *p* < 0.0001), but not contemporary (*p* > 0.05), pathogen prevalence was a significant positive predictor of allelic frequency of the serotonin transporter gene. Nations with a higher historical prevalence of disease-causing pathogens showed a higher prevalence of S allele carriers (see electronic supplementary material, figure S2). Additionally, consistent with prior findings from Fincher *et al*. (2008), across 29 nations, global historical (*β* = 0.69, *p* < 0.0001) and contemporary (*β* = 0.51, *p* < 0.005) pathogen prevalence was a significant positive predictor of collectivism. We subsequently conducted a mediation regression where historical pathogen prevalence and S allele frequency were included as predictors of cultural values of individualism–collectivism across 29 nations. Results of this mediation regression analysis showed that S allele frequency remained a reliable predictor (*β* = 0.45, *p* < 0.007), and the effect of global historical pathogen prevalence decreased significantly (from *r*(29) = 0.72 to *r*(29) = 0.42; Sobel test *Z* = 2.28, *p* < 0.02) (figure 4). Taken together, these results indicate that historical, but not contemporary, pathogen prevalence predicts cultural variability of individualism–collectivism due to increased S allelic frequency of the serotonin transporter gene.

## 4. Discussion

Here, we demonstrate for the first time a robust association between cultural values of individualism–collectivism and allelic frequency of the serotonin transporter gene, controlling for associated economic and disease factors. Geographical regions characterized by cultural collectivism exhibit a greater prevalence of S allele carriers of the serotonin transporter gene, even when cultural regions rather than nations served as the unit of analysis. Additionally, we show that global variability in historical pathogen prevalence predicts global variability in individualism–collectivism owing to genetic selection of the S allele of the serotonin transporter gene in regions characterized by high collectivism. Importantly, we also reveal a novel and surprising negative association between individualism–collectivism, frequency of S allele carriers of the serotonin transporter gene and global prevalence of anxiety and mood disorder. Across nations, both collectivism and allelic frequency of the S allele negatively predict global prevalence of anxiety and mood disorders. Critically, our results further indicate that greater population frequency of S allele carriers is associated with decreased prevalence of anxiety and mood disorders due to increased cultural collectivism.

The current findings suggest a novel demonstration of culture–gene coevolution of human behaviour. Emphasizing social norms that increase social harmony and encourage giving social support to others, collectivism serves an ‘anti-psychopathology’ function by creating an ecological niche that lowers the prevalence of chronic life stress, protecting genetically susceptible individuals from environmental pathogens known to trigger negative emotion and psychopathology. These findings complement notions that cultural values of individualism and collectivism are adaptive and by-products of evolution, more broadly. For instance, recent evidence suggests that cultural values of collectivism also serve an ‘anti-pathogen defence’ whereby behavioural manifestations of collectivism, such as conformity and parochialism, function as buffers against the transmission and increased prevalence of disease-causing pathogens (e.g. malaria, typhus and tuberculosis) (Fincher *et al*. 2008). Our results provide novel evidence that geographical regions characterized by collectivistic cultural norms have a higher historical and contemporary prevalence of infectious diseases due, at least partially, to genetic selection of S allele carriers (Fincher *et al*. 2008). Taken together, these findings dovetail nicely as two examples of how cultural values serve adaptive functions by tuning societal behaviour so that social and environmental risk factors are reduced and physical and mental health of group members is maintained. Importantly, in the current study, we found that population frequency of the serotonin transporter gene was a singular predictor of cultural values of individualism–collectivism across nations, even when controlling for historical and contemporary pathogen prevalence. Hence, our findings illustrate that gene frequency plays a unique role in explaining global variation in the adoption of cultural norms and is fundamental to any comprehensive understanding of culture.

A central claim of culture–gene coevolutionary theory is that once cultural traits are adaptive, it is likely that genetic selection causes refinement of the cognitive and neural architecture responsible for the storage and transmission of those cultural capacities (Boyd & Richerson 1985). Extending this logic to the current findings, we speculate that S and L allele carriers of the serotonin transporter gene may possess at least two kinds of information processing biases in the mind and brain that enhance their ability to store and transmit collectivistic and individualistic cultural norms, respectively. Affective biases in attention and cognition may serve as likely candidate information processing mechanisms involved in the storage and transmission of cultural values of individualism and collectivism. One possibility is that positive and negative information processing biases may serve to facilitate individualistic and collectivistic cultural norms. Recent behavioural evidence indicates that individuals carrying the S allele exhibit stronger attentional bias for negative words (Beevers *et al*. 2007) and pictures (Osinsky *et al*. 2008), whereas individuals carrying the L allele demonstrate a stronger attentional bias towards positive pictures and away from negative pictures (Fox *et al*. 2009). By extension, S allele carriers may be more likely to demonstrate negative cognitive biases, such as engage in narrow thinking and cognitive focus, which facilitate maintenance to collectivistic cultural norms of social conformity and interdependence, whereas L allele carriers may exhibit positive cognitive biases, such as open, creative thinking and greater willingness to take risks, which promote individualistic cultural norms of self-expression and autonomy (Isen *et al*. 1987; Fredrickson 2001). Future research in cultural psychology may examine whether or not cultural values of individualism–collectivism are associated with affective biases towards positive and negative information, respectively, and if so, the process by which affective biases in perception and cognition facilitate the storage and transmission of cultural values and culturally congruent behaviours (e.g. attending to others versus asserting one's self).

Neural activity within brain regions innervated by serotonergic neural pathways, such as the human amygdala, may serve as another likely information processing mechanism involved in the storage and transmission of cultural values of individualism and collectivism. For instance, recent evidence from imaging genetics demonstrates that individuals carrying the S allele show greater amygdala response to emotional stimuli (Hariri *et al*. 2002; Munafò *et al*. 2008), which is likely due to increased amygdala resting activation (Canli *et al*. 2005) and decreased functional coupling between the amygdala and subgenual cingulate gyrus (Pezawas *et al*. 2005), relative to individuals carrying the L allele. Recent cross-cultural neuroimaging evidence demonstrates cultural specificity in amygdala response to fear faces (Chiao *et al*. 2008) as well as modulation of medial prefrontal response during self-relevant processing as a function of individualistic and collectivistic cultural values (Chiao *et al*. 2009*a*,*b*). Future research in cultural neuroscience (Chiao in press) may investigate the extent to which cultural values of individualism–collectivism are associated with neural response within brain regions regulated by serotonergic neurotransmission, and if so, the process by which these activity within neural pathways supports the storage and transmission of cultural values and related behaviours.

The current evidence for culture–gene coevolution of individualism–collectivism and the serotonin transporter gene may provide further novel insight into the functional significance of endophenotypes associated with the serotonin transporter gene across cultural contexts. Both of the putative information processing mechanisms that facilitate the storage and transmission of cultural values of individualism and collectivism described above are considered intermediate phenotypes or endophenotypes of affective disorders (Canli *et al*. 2006; Caspi & Moffitt 2006). Individual differences in anxiety and depression are associated with robust selective attention (Ohman & Mineka 2001), as well as increased amygdala response (Bishop 2007), to negative information, even in normal populations. We suggest that these endophenotypes may confer varying degrees of advantage or disadvantage to individuals depending on the cultural context. For people living in collectivistic cultures, heightened selective attention and increased amygdala response to negative information may be advantageous to achieving collectivistic cultural norms, such as maintaining social harmony. For instance, greater vigilance to negative information may be useful for early detection of another person's anger or fear as well as foreshadowing and avoiding actions or interpersonal situations that may induce negative emotional states in others. Also, greater vigilance to negative information may encourage a stronger narrow thinking and cognitive focus, enabling one to effectively conform to social norms. By contrast, for people living in individualistic cultures, heightened selective attention and increased amygdala response to negative information may be disadvantageous to achieving individualistic cultural norms of self-expression and assertion of self-interests. For instance, greater vigilance to negative information may make one hesitant to express their thoughts and feelings in social contexts or behave in an assertive manner, making it difficult to form and maintain meaningful social relationships in individualistic societies, a social behaviour critical to reducing the risk of affective disorders for genetically susceptible individuals. At the same time, heightened biases for positive information may be advantageous in individualistic cultures. For instance, positive information biases have been shown to encourage creative thinking and openness to novelty and risk-taking (Isen *et al*. 1987; Fredrickson 2001), which may in turn encourage independent, assertive social behaviour and increase the likelihood of social connection with others. Hence, the functional utility of endophenotypes associated with the serotonin transporter gene may systematically vary as a function of cultural context. Future research is needed to further determine the role that endophenotypes play in the transmission and maintenance of cultural values, practices and beliefs.

A possible limitation of the current study is the reliance on cross-national epidemiological reports for estimates of mental illness prevalence, which may be vulnerable to response biases. For instance, individuals living in collectivistic nations, such as East Asia, are known to exhibit higher levels of stigma towards mental illness, relative to individuals living in individualistic nations, due to increased cultural pressures to save face and conform to social norms (Ng 1997). Hence, it is possible that decreased prevalence of mental illnesses in East Asia may be due, in part, to response biases. Importantly, in the current study, divergent validity analyses indicated that cultural values and allelic frequency of the serotonin transporter gene predicted global prevalence of anxiety and mood disorders, but not impulse control and substance abuse. If response biases were evident in the current cross-national estimates of mental health disorder prevalence, it is likely that they would influence the cross-national prevalence estimates of all of the disorders, not only anxiety and mood disorders. Hence, we suggest that the observed relationship between cultural values, gene frequency of the serotonin transporter gene and affective disorders is not likely due to response biases.

Understanding the aetiology of mental health disorders, such as anxiety and mood disorders, is vital to relieving the substantial emotional and economic burdens associated with their onset and treatment (Kessler & Ustun 2008). Behavioural genetics studies examining the association between polymorphisms of the serotonin transporter gene and affective disorders (Uher & McGuffin 2008) as well as the association between environmental interactions with the serotonin transporter gene and affective disorders (Munafo *et al*. 2009; Risch *et al*. 2009) within a given population often produce inconsistent results, suggesting a more complex path from gene to disease. The importance of considering GxE interactions in understanding the aetiology of complex psychiatric disorders has become more widely acknowledged (Caspi & Moffitt 2006; Canli & Lesch 2007; Munafo *et al*. 2009), yet the association between specific cultural and genetic factors underlying affective disorders across human populations has been largely unexplored until now. The present work provides macro-scale evidence for how cultural values play an adaptive role in buffering genetically vulnerable populations from a potentially heightened epidemiological prevalence of mental health disorders. Our cross-population findings complement recent evidence from a within-population study conducted in urban Brazil showing an adaptive benefit of cultural values in buffering genetically vulnerable individuals from depressive symptoms (Dressler *et al*. 2009). Taken together, these studies underscore the utility of incorporating cultural traits, such as individualism–collectivism, in macro- (e.g. cross-population) and micro-scale (e.g. within-population) models of GxE factors underlying complex affective disorders and the importance of culture–gene coevolutionary theory for understanding typical and atypical human behaviour, more broadly construed.
